# Global Prevalence, Regional Distribution, and Demographic Patterns of Schistosomal Appendicitis: A Systematic Review and Meta-Analysis

**DOI:** 10.7759/cureus.95872

**Published:** 2025-11-01

**Authors:** Younis Al-Mufargi, Nisreen Al-Busaidi, Haya Al Maamari, Amina Al-Hatmi, Badriya Al-Mammari, Jinan Al-Shukaili, Mohammed Al-Subhi, Ahmed Abd Elrahman, Yaqoob AlSawafi

**Affiliations:** 1 General Surgery, Medical City for Military and Security Services, Muscat, OMN; 2 General Practice, Medical City for Military and Security Services, Muscat, OMN; 3 Medicine and Health Sciences, Sultan Qaboos University, Muscat, OMN; 4 Medicine, University of Aberdeen, Scotland, GBR

**Keywords:** appendectomy, global prevalence, histopathology, meta-analysis, schistosomal appendicitis, schistosomiasis

## Abstract

The prevalence of schistosomal appendicitis (SA) remains uncertain due to its infrequent occurrence in medical practice. This systematic review and meta-analysis aimed to determine the global prevalence and distribution patterns of SA. Following the Preferred Reporting Items for Systematic Reviews and Meta-Analyses (PRISMA) guidelines, five databases were searched up to June 2025. Eligible studies were observational cohorts reporting histologically confirmed cases of SA from appendectomy specimens. Two independent reviewers conducted data extraction, and study quality was assessed using the Joanna Briggs Institute (JBI) checklist for prevalence studies. A random-effects meta-analysis was performed to estimate pooled prevalence, with subgroup analyses by endemicity, region, income level, and study period. Twenty-four studies comprising 41,301 appendectomy specimens were included. The global pooled prevalence of SA was 1.84% (95% CI: 1.13%-2.71%). Prevalence was higher in endemic regions (2.67%, 95% CI: 1.81%-3.69%) than in non-endemic regions (0.16%, 95% CI: 0.10%-0.24%). Lower-middle-income countries had the highest prevalence (2.66%, 95% CI: 1.94%-3.47%), followed by upper-middle-income (2.10%, 95% CI: 0.55%-4.58%) and high-income countries (0.59%, 95% CI: 0.24%-1.08%). Regionally, Africa had the highest pooled prevalence (3.22%, 95% CI: 1.69%-5.18%), followed by Asia (1.01%, 95% CI: 0.39%-1.91%). Among 247 histologically confirmed cases, 91.92% (95% CI: 76.22%-100.00%) showed appendiceal inflammation. A temporal trend indicated rising SA prevalence after 2015, and a male predominance was noted, with a male-to-female ratio of 2.78:1. Heterogeneity among studies was high (I² = 96.7%), and funnel plot analysis suggested potential publication bias. Despite declining global incidence, SA remains a significant cause of appendicitis in endemic areas. Routine histopathological examination of appendectomy specimens is recommended, alongside sustained control and elimination efforts to reduce disease burden.

## Introduction and background

Schistosomiasis is a disease that continues to impact the lives of over 200 million people globally, especially in parts of sub-Saharan Africa, Southeast Asia, and South America (World Health Organization) [1]. It is caused by parasitic worms known as *Schistosoma*, and the infection usually targets the urinary and digestive systems. However, sometimes, the parasite’s eggs end up in unexpected places - such as the appendix - leading to a lesser-known complication called schistosomal appendicitis (SA) [2]. Early case series by Satti et al. [3] first described the incidental and inflamed presence of schistosomal ova in appendiceal tissue, laying the foundation for recognizing SA as a distinct clinical entity.

In SA, the body reacts to the trapped eggs with inflammation, a flood of immune cells such as eosinophils and, over time, thickening or even hardening of the appendix wall [3]. While doctors in regions where schistosomiasis is common are more likely to recognize it, SA is often missed in countries where the disease is rare and where appendix tissue is not routinely examined after surgery. Reported cases vary a lot - showing up in less than 1% of appendectomies in non-endemic areas, but in over 5% in regions where schistosomiasis is widespread [4-6].

An earlier review by Zacarias et al. [7] provided a foundational summary of existing knowledge on SA, but it had notable limitations, including a smaller evidence base, a lack of recent studies, and a limited analysis of how SA prevalence varies by geography, income level, or endemicity. To address these gaps, we conducted a more comprehensive and up-to-date systematic review and meta-analysis, incorporating 24 studies and data from over 41,300 appendectomy cases across five WHO regions. Our analysis not only offers a more accurate estimate of global SA prevalence but also examines its variation by region, national income classification, endemicity, and temporal trends. Additionally, we explore demographic patterns and clinical presentations associated with SA. This expanded perspective aims to enhance understanding of SA within the broader context of global health and to highlight areas where increased awareness and research are needed.

## Review

Materials and methods

Study Design

This systematic review and meta-analysis followed the Preferred Reporting Items for Systematic Reviews and Meta-Analyses (PRISMA) guidance. The primary objective was to estimate the prevalence of histologically confirmed SA among appendectomy specimens. Secondary objectives were to (a) estimate pooled subgroup prevalences by endemicity, WHO region, income level, and study period, and (b) to compare prevalence by sex and inflamed appendix versus incidental findings of schistosoma.

Eligibility Criteria

Studies were eligible for inclusion if they were observational in design (either retrospective or prospective) and involved human participants who underwent appendectomy. To be included, studies had to report both (1) the number of appendectomy specimens examined histologically and (2) the number of specimens found to have SA, defined by the presence of *Schistosoma *ova within appendiceal tissue. No language restrictions were applied during the search. Conference abstracts, theses, and unpublished data were excluded due to a lack of peer review and insufficient extractable data. In addition, no restrictions were placed on age, sex, or demographic background of study populations.

Studies were excluded if they were case reports or case series involving fewer than 30 patients. Reviews, editorials, and conference abstracts without available full texts were also excluded. Additionally, studies were excluded if they lacked histopathological confirmation of schistosomiasis in the appendix, did not provide extractable numerators and denominators necessary for calculating prevalence, or were unable to differentiate SA from other parasitic or non-parasitic appendiceal conditions. In instances where overlapping cohorts were identified across publications, the most complete or the most recent report was retained for inclusion.

Search Strategy

We searched PubMed, Scopus, ScienceDirect, EBSCOhost, and Wiley Online Library from inception to June 30, 2025, without language or geographic restrictions. The search combined controlled vocabulary and keywords linking schistosomiasis to appendicitis and histopathology: (schistosomiasis OR schistosoma OR schistosome) AND (appendicitis OR appendix OR appendectomy) AND (histopathology OR histological OR pathology OR biopsy). Reference lists of eligible articles and related reviews were screened to identify additional studies. Records were imported into Rayyan for de-duplication and blinded screening.

Study Selection

**Figure 1:**
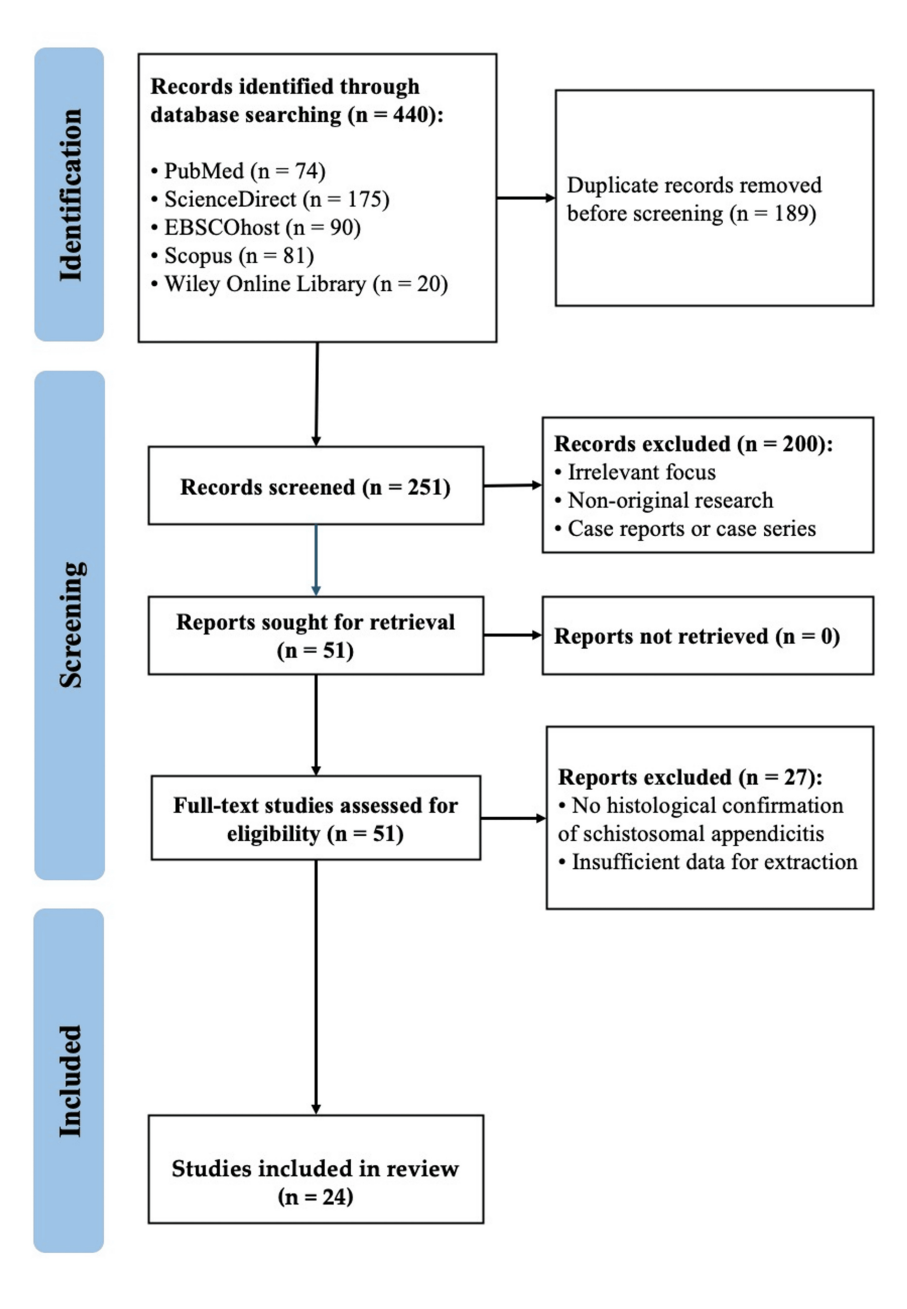
PRISMA Flow Diagram of the Study Selection PRISMA: Preferred Reporting Items for Systematic Reviews and Meta-Analyses

Two reviewers independently screened titles/abstracts and then full texts against eligibility criteria. Disagreements were resolved by consensus or adjudication by a third reviewer. The study selection process is depicted in a PRISMA flow diagram (Figure 1).

Data Extraction

Two reviewers independently extracted data using a standardized form: first author, year, country, WHO region, study period, design, sample size, number of SA cases, age distribution, sex distribution, and diagnostic approach. Discrepancies were reconciled by consensus. Studies with incomplete demographic data were retained if histological confirmation of SA and extractable prevalence data were available.

Quality Assessment

Due to the inclusion of predominantly observational studies, quality was assessed using a simplified version of the Joanna Briggs Institute (JBI) checklist for prevalence studies, evaluating aspects such as sampling methods, case definition, diagnostic criteria, and completeness of reporting. The quality assessment was conducted by two independent reviewers. No studies were excluded based on quality scores, but sensitivity analyses were applied.

Data Synthesis and Statistical Analysis

A random-effects model (DerSimonian and Laird) with double arcsine transformation was used to pool prevalence estimates, and heterogeneity was assessed using the I² statistic and Cochran’s Q test. The primary outcome was the pooled prevalence of SA, expressed as a proportion of confirmed cases among total appendectomy specimens, with 95% confidence intervals (CI).

Subgroup analyses were performed based on several factors, including geographic continent (e.g., Africa, Asia) and whether regions were endemic or non-endemic, country income classification, the time period during which each study was conducted, sex distribution, and histopathological findings. Additionally, a time-trend analysis was carried out to assess changes in prevalence across different decades. Heterogeneity was assessed using the I² statistic and chi-squared test, with I² values >75% indicating high heterogeneity.

All statistical analyses were conducted using R software (4.5.1; 2025-06-13; R Development Core Team, Vienna, Austria) in RStudio (version 2023.12.1+402; RStudio Team, Boston, MA).

Outcomes Measured

The primary outcome was the prevalence of SA among appendectomy specimens. Secondary outcomes were odds ratios comparing (i) males versus females and (ii) inflamed appendix versus incidental schistosomiasis. 

Protocol and Registration

No PROSPERO registration was undertaken; however, the review followed a predefined protocol that guided all stages of screening, extraction, and synthesis. This protocol is available upon request for transparency.

Results

A total of 440 records were identified through systematic database searching, including PubMed, ScienceDirect, EBSCOhost, Scopus, and Wiley Online Library. After removing 189 duplicates, 251 records were screened, and 200 were excluded based on relevance, originality, and study type. Of the 51 full-text articles assessed for eligibility, 27 were excluded due to a lack of histological confirmation or insufficient data for extraction. Ultimately, 24 studies met the inclusion criteria and were included in the final meta-analysis (Figure 1). The summary characteristics of the included studies are shown in Table 1.

**Table 1:** Overview of the Study Characteristics Included in the Review (n = 24)

Study (Author, Year)	Country	Study Design	Period	SA (n)	Total (n)	Prevalence (%)	Age (mean/range)	Sex (M:F)	Species Identified
Abo-Alhassan et al., 2016 [8]	Kuwait	Retrospective	2007–2011	8	3012	0.27	32.8 (24–42)	8:00	Not stated
Abu-Eshy et al., 1995 [4]	Saudi Arabia	Retrospective	1987–1993	64	4708	1.36	31.9 ± 8.6	Not stated	Not stated
Adebamowo et al., 1991 [9]	Nigeria	Retrospective	1980–1989	15	627	2.39	10–44	All male	S. haematobium
Adisa et al., 2008 [10]	Nigeria	Retrospective	Not stated	22	956	2.30	27.4 ± 8.2	39:07:00	Not stated
Ahmed et al., 2014 [6]	Nigeria	Retrospective	1991–2012	30	1,464	2.05	13–55	26:04:00	S. haematobium, S. mansoni
Ahmed et al., 2017 [11]	Egypt	Retrospective	2013–2016	1	30	3.33	35–50	30:00:00	S. mansoni
Al-Kraida et al., 1988 [12]	Saudi Arabia	Retrospective	1985–1986	15	1920	0.78	31.6 avg.	12:03	S. haematobium, S. mansoni
Amer et al., 2018 [13]	Egypt	Cross-sectional	2015–2016	1	65	1.54	6–60	Not stated	S. mansoni
Badmos et al., 2006 [14]	Nigeria	Retrospective	1991–2004	35	843	4.15	13–62 (median 25)	26:09:00	Not stated
Botes et al., 2015 [15]	South Africa	Retrospective	2009–2013	31	304	10.20	5–86 (mean 23.5)	14:17	Not stated
Dincel et al., 2008 [16]	Turkey	Retrospective	Not stated	2	1970	0.10	Not stated	Not stated	S. mansoni
Duduyemi et al., 2011 [5]	Nigeria	Retrospective	2003–2007	7	293	2.39	Mostly <40	6:02	S. haematobium
Duvie et al., 1987 [17]	Nigeria	Prospective	Not stated	32	518	6.18	Not stated	Not stated	S. haematobium
Elfaedy et al., 2016 [18]	Egypt	Prospective	2014–2016	8	4012	0.20	23.8 mean	Not stated	Not stated
Hedya et al., 1996 [19]	Sudan	Retrospective	2014–2018	3	243	1.23	7–47	10:11	S. mansoni
Hodasi et al., 1988 [20]	Ghana	Retrospective	Not stated	76	2584	2.94	5–60 (mean 14)	57:19:00	S. haematobium
Karatepe et al., 2009 [21]	Turkey	Retrospective	2001–2008	6	5100	0.12	Not stated	Not stated	S. mansoni
Nandipati et al., 2007 [22]	USA	Retrospective	Not stated	3	1690	0.18	29.3 ± 9.5	Not stated	S. haematobium
Satti et al., 2012 [3]	Sudan	Retrospective	1990–1996	26	1600	1.63	12–42	18:07	S. mansoni
Valluru et al., 2019 [23]	China	Case-control	Not stated	50	557	8.98	>50 in SA group	Not stated	S. japonicum
Wang et al., 2025 [24]	China	Retrospective	2013–2023	136	5554	2.45	SA: 61.7 ± 15.3	Not stated	S. japonicum
Zacarias et al., 2005 [7]	Mozambique	Retrospective	Not stated	19	145	13.10	12–35	2:01	S. haematobium
Zaghlool et al., 2015 [25]	Saudi Arabia	Retrospective	2012–2014	2	1506	0.13	12 (mean)	1:01	S. mansoni
Zakaria et al., 2013 [26]	Saudi Arabia	Retrospective	2001–2010	8	1600	0.50	2–18	Mixed	S. mansoni

**Figure 2:**
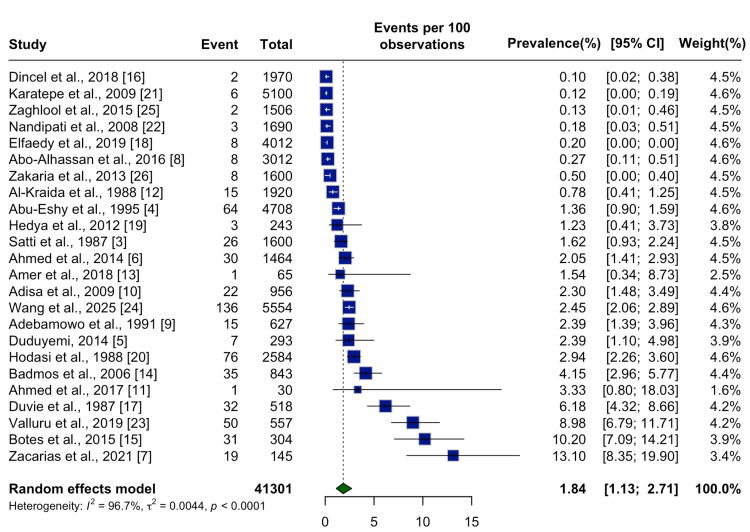
Forest Plot of the Pooled Global Prevalence of Schistosomal Appendicitis Forest plot showing the pooled prevalence of schistosomal appendicitis across 24 studies, using a random effects model. Studies included Dincel et al., 2018 [16]; Karatepe et al., 2009 [21]; Zaghlool et al., 2015 [25]; Nandipati et al., 2008 [22]; Elfaedy et al., 2019 [18]; Abo-Alhassan et al., 2016 [8]; Zakaria et al., 2013 [26]; Al-Kraida et al., 1988 [12]; Abu-Eshy et al., 1995 [4]; Hedya et al., 2012 [19]; Satti et al., 1987 [3]; Ahmed et al., 2014 [6]; Amer et al., 2018 [13]; Adisa et al., 2009 [10]; Wang et al., 2025 [24]; Adebamowo et al., 1991 [9]; Duduyemi, 2014 [5]; Hodasi et al., 1988 [20]; Badmos et al., 2006 [14]; Ahmed et al., 2017 [11]; Duvie et al., 1987 [17]; Valluru et al., 2019 [23]; Botes et al., 2015 [15]; Zacarias et al., 2021 [7].

The pooled global prevalence of SA was estimated at 1.84% (95% CI: 1.13%-2.71%) across a combined total of 41,301 appendectomy specimens, based on a random effects model (Figure 2). Substantial heterogeneity was observed among the studies (I² = 96.7%; p < 0.0001), indicating considerable variability across regions and methodologies.

**Figure 3:**
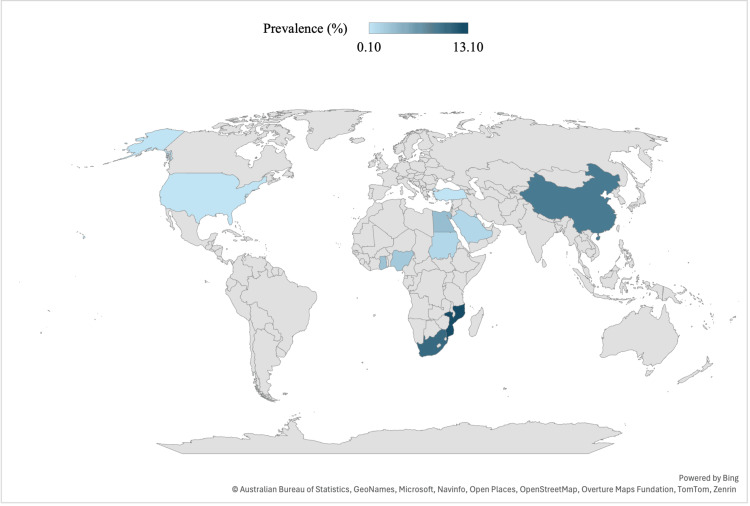
Spatial Epidemiology of Schistosomal Appendicitis: A Country-Level Analysis Image Credits: Younis Al-Mufargi, MD. Figure created using Microsoft Excel.

Spatial analysis demonstrated a distinct geographical distribution of SA. Higher prevalence was particularly evident in parts of sub-Saharan Africa, such as Mozambique and South Africa, and East Asia, notably China. In contrast, countries in North America and Europe showed much lower prevalence rates (Figure 3). These findings align with known endemic zones of schistosomiasis, supporting a geographic correlation.

**Figure 4:**
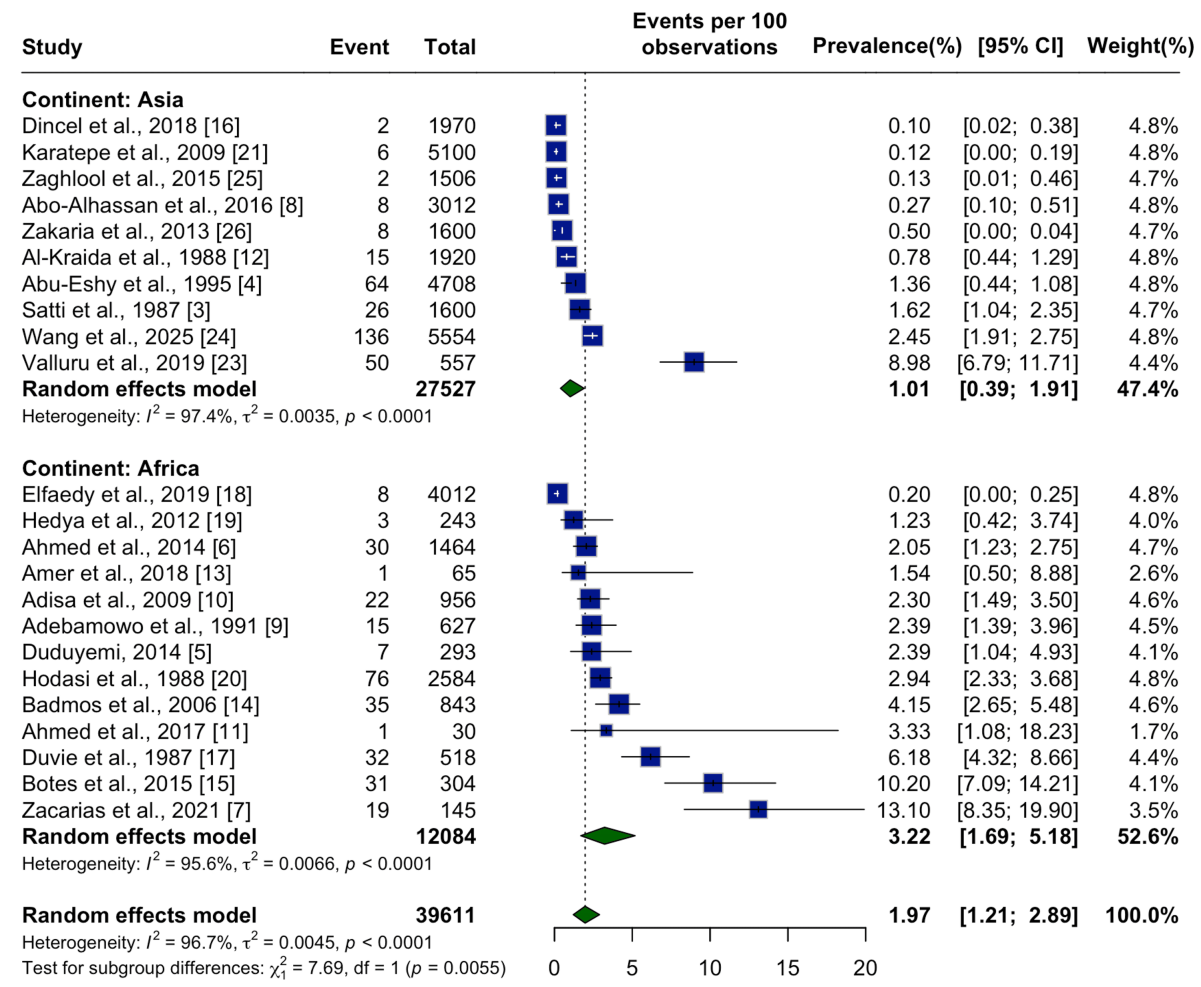
Subgroup Meta-Analysis by Continent Subgroup analysis by continent (Asia vs. Africa) showing the prevalence of Schistosomal appendicitis following laparoscopic procedures, based on 23 studies. A random effects model was used for each subgroup and overall. Studies from Asia: Dincel et al., 2018 [16]; Karatepe et al., 2009 [21]; Zaghlool et al., 2015 [25]; Abo-Alhassan et al., 2016 [8]; Zakaria et al., 2013 [26]; Al-Kraida et al., 1988 [12]; Abu-Eshy et al., 1995 [4]; Satti et al., 1987 [3]; Wang et al., 2025 [24]; Valluru et al., 2019 [23]. Studies from Africa: Elfaedy et al., 2019 [18]; Hedya et al., 2012 [19]; Ahmed et al., 2014 [6]; Amer et al., 2018 [13]; Adisa et al., 2009 [10]; Adebamowo et al., 1991 [9]; Duduyemi, 2014 [5]; Hodasi et al., 1988 [20]; Badmos et al., 2006 [14]; Ahmed et al., 2017 [11]; Duvie et al., 1987 [17]; Botes et al., 2015 [15]; Zacarias et al., 2021 [7]. Note: A significant difference was observed between continents (p = 0.0055), with higher pooled prevalence in African studies (3.22%) compared to Asian studies (1.01%).

Subgroup analyses revealed significant differences by continent. Studies from Africa showed a substantially higher pooled prevalence of 3.22% (95% CI: 1.69%-5.18%) compared to 1.01% (95% CI: 0.39%-1.91%) in Asia. The difference was statistically significant (p = 0.0055), suggesting a continent-level disparity in SA burden (Figure 4).

**Figure 5:**
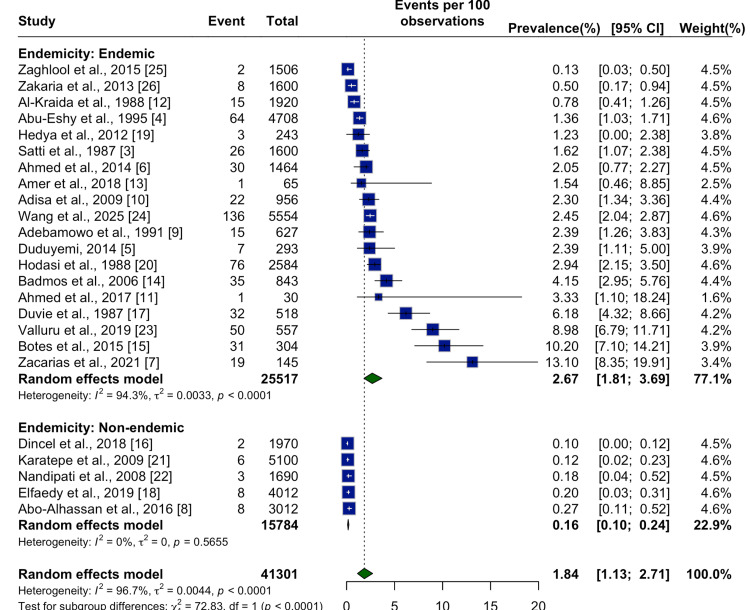
Subgroup Meta-Analysis by Endemicity Status Forest plot comparing the prevalence of appendicular schistosomiasis in endemic versus non-endemic regions across 24 studies. A random effects model was applied within each group and overall. The pooled prevalence was significantly higher in endemic settings (2.67% (1.81-3.69)) compared to non-endemic settings (0.16% (0.10-0.24)), with strong statistical evidence for subgroup difference (p < 0.0001). In endemic regions, studies such as Zaghlool et al. [25], Zakaria et al. [26], and Al-Kraida et al. [12] reported relatively low to moderate prevalence (ranging from 0.13% to 0.78%), while Abu-Eshy et al. [4], Hedya et al. [19], and Satti et al. [3] reported higher values, between 1.2% and 1.6%. A wide range was observed among later studies, including Ahmed et al. [6], Amer et al. [13], Adisa et al. [10], and Wang et al. [24], with prevalence estimates from 1.5% up to 2.5%. Several studies—Adebamowo et al. [9], Duduyemi [5], Hodasi et al. [20], and Badmos et al. [14]—reported prevalence exceeding 2%, while Ahmed et al. [11], Duvie et al. [17], Valluru et al. [23], Botes et al. [15], and Zacarias et al. [7] observed the highest rates, up to 13.1%. In contrast, studies conducted in non-endemic regions, including Dincel et al. [16], Karatepe et al. [21], Nandipati et al. [22], Elfaedy et al. [18], and Abo-Alhassan et al. [8], consistently reported very low prevalence, all below 0.3%, with minimal heterogeneity (I² = 0%).

Further stratification by endemicity status highlighted stark contrasts. In endemic regions, the pooled prevalence was 2.67% (95% CI: 1.81%-3.69%), whereas non-endemic regions reported a prevalence of only 0.16% (95% CI: 0.10%-0.24%), with a statistically significant subgroup difference (p < 0.0001) (Figure 5). These findings reinforce the strong association between schistosomal exposure and appendiceal involvement.

**Figure 6:**
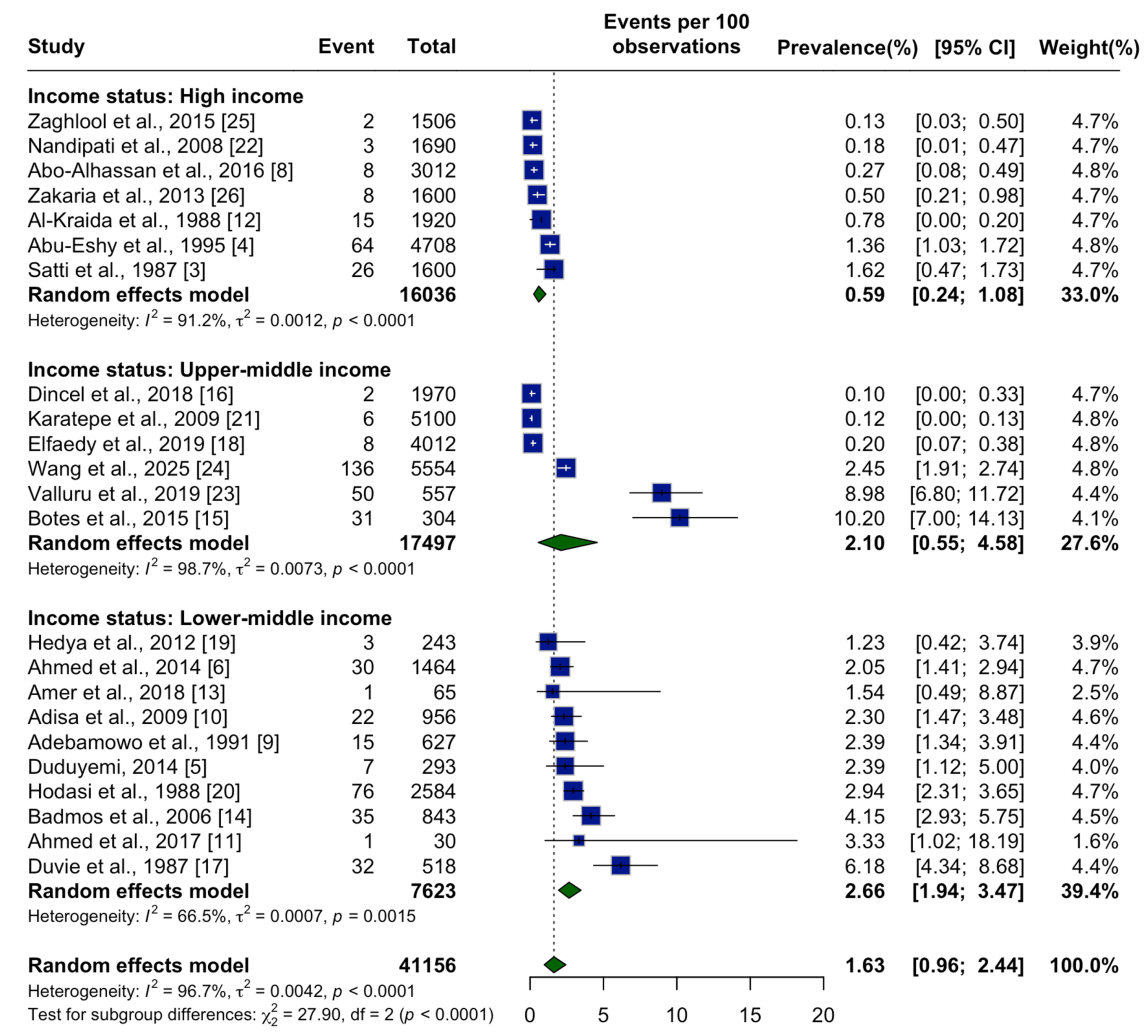
Subgroup Meta-Analysis by Country Income Level Subgroup analysis by national income status, showing the pooled prevalence of schistosomal appendicitis. Income status was categorized based on World Bank classification at the time of study publication. A random effects model was used within each income group and overall. High-income countries: Zaghlool et al., 2015 [25]; Nandipati et al., 2008 [22]; Abo-Alhassan et al., 2016 [8]; Zakaria et al., 2013 [26]; Al-Kraida et al., 1988 [12]; Abu-Eshy et al., 1995 [4]; Satti et al., 1987 [3]. Upper-middle-income countries: Dincel et al., 2018 [16]; Karatepe et al., 2009 [21]; Elfaedy et al., 2019 [18]; Wang et al., 2025 [24]; Valluru et al., 2019 [23]; Botes et al., 2015 [15]. Lower-middle-income countries: Hedya et al., 2012 [19]; Ahmed et al., 2014 [6]; Amer et al., 2018 [13]; Adisa et al., 2009 [10]; Adebamowo et al., 1991 [9]; Duduyemi, 2014 [5]; Hodasi et al., 1988 [20]; Badmos et al., 2006 [14]; Ahmed et al., 2017 [11]; Duvie et al., 1987 [17]. The overall pooled prevalence was 1.63% (0.96%, 2.44%), with higher rates in lower-middle-income countries (2.66%) compared to upper-middle (2.10%) and high-income (0.59%) settings.

Analysis by country income level also demonstrated disparities. High-income countries had the lowest prevalence at 0.59% (95% CI: 0.24%-1.08%), while upper-middle and lower-middle income countries had higher rates of 2.10% (95% CI: 0.55%-4.58%) and 2.66% (95% CI: 1.94%-3.47%), respectively (Figure 6). This gradient may reflect differences in sanitation, water exposure, and schistosomiasis control programs.

**Figure 7:**
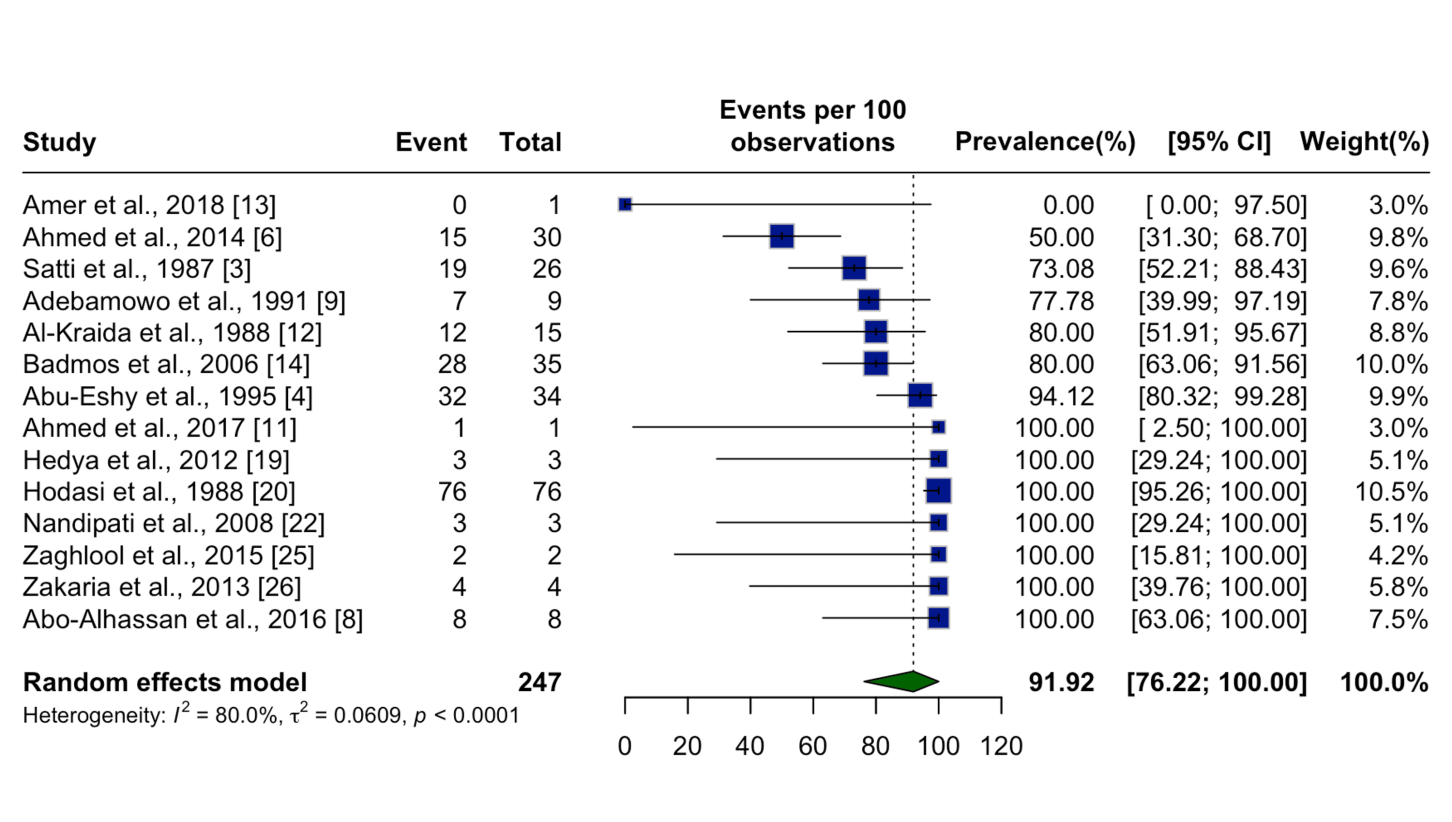
Proportion of Inflamed Appendices Among Schistosomal Cases Forest plot showing the pooled prevalence of appendectomies in cases involving inflamed appendix across 14 studies. A random effects model was applied. The overall pooled prevalence was 91.92% (76.22%, 100.00%), indicating a substantially increased risk under inflamed conditions. Studies included: Amer et al., 2018 [13]; Ahmed et al., 2014 [6]; Satti et al., 1987 [3]; Adebamowo et al., 1991 [9]; Al-Kraida et al., 1988 [12]; Badmos et al., 2006 [14]; Abu-Eshy et al., 1995 [4]; Ahmed et al., 2017 [11]; Hedya et al., 2012 [19]; Hodasi et al., 1988 [20]; Nandipati et al., 2008 [22]; Zaghlool et al., 2015 [25]; Zakaria et al., 2013 [26]; Abo-Alhassan et al., 2016 [8]. Note: The heterogeneity was significant (I² = 80.0%, p < 0.0001), reflecting variation among studies, likely due to differences in population characteristics or definitions of "inflamed."

Histopathological analysis of 247 confirmed cases revealed that 91.92% (95% CI: 76.22%-100.00%) of SA cases had visible schistosome ova on microscopic examination and an inflamed appendix (Figure 7).

**Figure 8:**
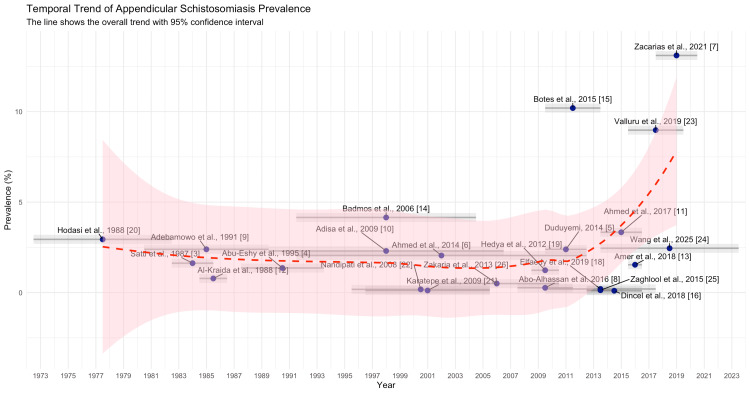
Temporal Trend of Schistosomal Appendicitis Prevalence Temporal trend in the reported prevalence of appendicular schistosomiasis across 23 studies published between 1987 and 2025. Each data point represents the prevalence reported in an individual study, labeled with the author and year. The red dashed line indicates the fitted trend over time, and the shaded region represents the 95% confidence interval. Earlier studies, such as those by Satti [3], Hodasi [20], Al-Kraida [12], Adebamowo [9], and Abu-Eshy [4], reported relatively low and stable prevalence rates. Throughout the 2000s, similar findings were observed in studies by Nandipati [22], Badmos [14], Karatepe [21], Adisa [10], Ahmed [6], and Hedya [19]. However, more recent publications—particularly those by Duduyemi [5], Zakaria [26], Elfaedy [18], Abo-Alhassan [8], Zaghlool [25], Dincel [16], Amer [13], Ahmed [11], Valluru [23], Botes [15], Wang [24], and Zacarias [7]—demonstrate an upward trend, with some reporting prevalence rates exceeding 10%.

Temporal analysis of prevalence rates showed a biphasic trend. A slight decline was observed from the 1980s to the early 2000s, likely due to improved public health interventions. However, recent studies demonstrated an increasing trend in prevalence after 2010, with the highest values reported by Zacarias et al. [7] and Wang et al. [24], suggesting a resurgence or improved detection in recent years (Figure 8).

**Figure 9:**
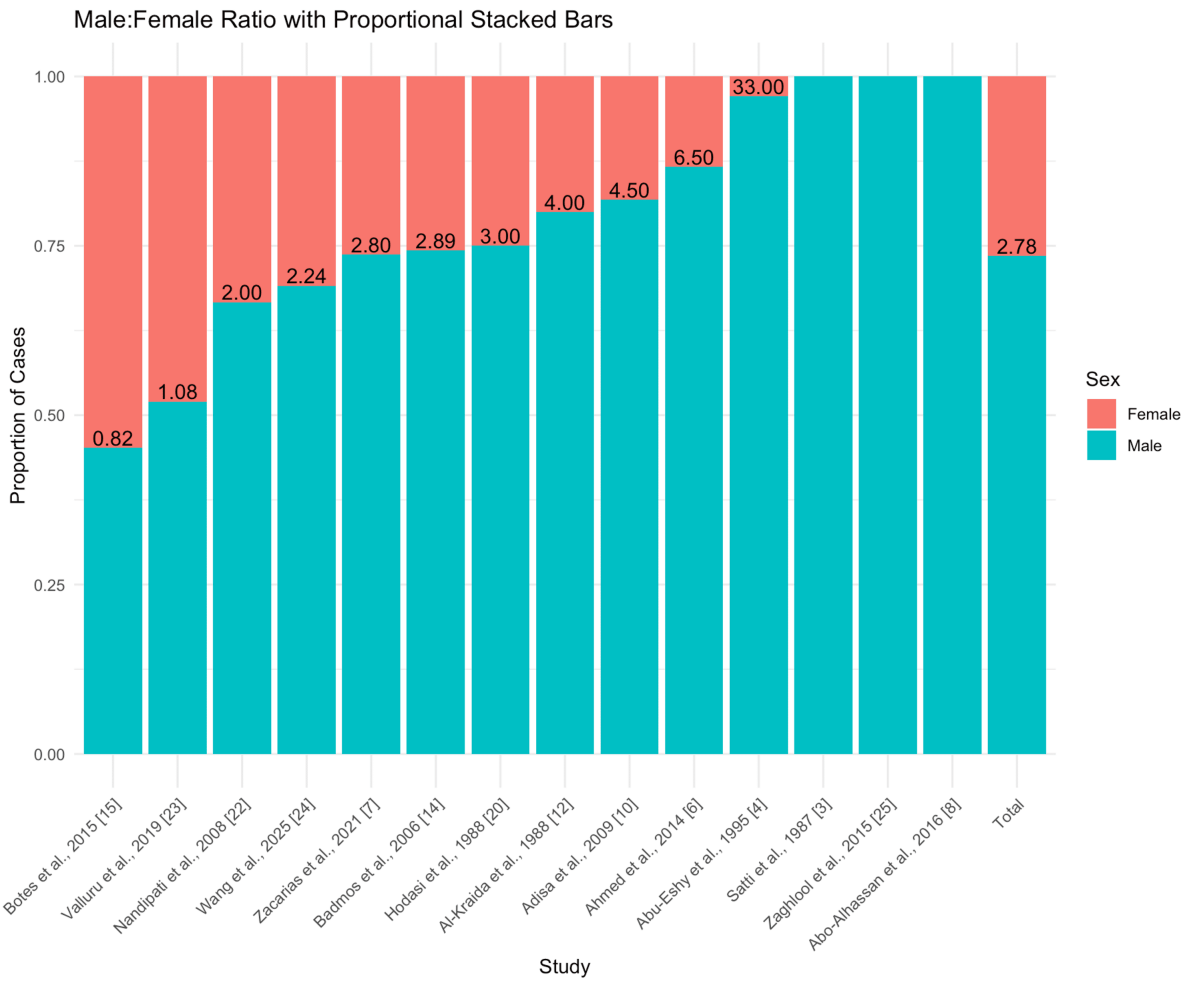
Male-to-Female Ratio in Schistosomal Appendicitis Cases Proportional stacked bar chart illustrating the male-to-female ratio of appendicular schistosomiasis cases reported across studies. Each bar represents the proportion of male (blue) and female (red) patients within a study, with the calculated male:female ratio labeled above. The rightmost bar displays the combined proportions across all included studies. Most studies reported a male predominance. For example, Zaghlool et al. [25] and Abo-Alhassan et al. [8] reported particularly high male:female ratios of 33.0:1 and 4.5:1, respectively, while Satti et al. [3] and Abu-Eshy et al. [4] observed ratios of 4.0:1 and 4.5:1. Moderate male bias was noted in studies such as Ahmed et al. [6], Adisa et al. [10], Al-Kraida et al. [12], and Hodasi et al. [20], with ratios ranging from 2.8:1 to 3.0:1. A more balanced sex distribution was found in Badmos et al. [14], Zacarias et al. [7], Wang et al. [24], and Nandipati et al. [22], with male:female ratios close to 2.0:1 or below. Near-equal or female-predominant distributions were reported by Valluru et al. [23] and Botes et al. [15], with ratios of 1.08:1 and 0.82:1, respectively.

Sex distribution analysis across eligible studies revealed a consistent male predominance, with an overall male-to-female ratio of 2.78:1. Some studies reported extremely skewed ratios, such as 33:1 (Abo-Alhassan et al. [8]), indicating potential sex-based differences in exposure to contaminated water or occupational risks (Figure 9).

Visual inspection of the funnel plot revealed noticeable asymmetry, suggesting the presence of publication bias or small-study effects. Several smaller studies reported higher prevalence rates and were located outside the pseudo-95% confidence region, further supporting the likelihood of selective reporting.

Discussion

This systematic review and meta-analysis provide the most comprehensive synthesis of SA to date. Across 24 studies encompassing 41,301 appendectomy specimens, we identified a pooled prevalence of 1.84% (95% CI: 1.13%-2.71%). These findings confirm that while SA is relatively uncommon globally, it remains a clinically relevant and regionally variable cause of appendicitis.

Geographically, Africa demonstrated the highest pooled prevalence (3.22%), with most cases reported from endemic regions including Nigeria, Ghana, Sudan, and Mozambique. This aligns with the ongoing high transmission of schistosomiasis across sub-Saharan Africa [1]. The Eastern Mediterranean Region, although contributing the largest number of studies, showed a lower crude prevalence (1.7%), likely reflecting the impact of national schistosomiasis control programs, particularly in Egypt, where transmission has markedly declined since the implementation of large-scale control efforts [27,28]. In contrast, Asia reported a pooled prevalence of 1.01%, and non-endemic regions - including parts of Europe, the Americas, and the Western Pacific - showed much lower rates, consistent with minimal local transmission and the detection of cases primarily among migrants or travelers from endemic areas [2].

The prevalence of SA was significantly higher in endemic countries (2.67%) compared to non-endemic settings (0.16%), underscoring the direct relationship between local schistosomiasis transmission and appendiceal involvement. Similarly, countries classified as lower-middle-income by the World Bank exhibited the highest pooled prevalence (2.66%), whereas high-income countries had a markedly lower burden (0.59%). These patterns highlight the persistent impact of socioeconomic and environmental determinants such as inadequate water and sanitation infrastructure and limited access to preventive chemotherapy [29].

Importantly, temporal trend analysis revealed a non-linear but increasing prevalence over time, particularly in studies published after 2015. This may reflect better histopathological diagnostic capabilities, increased reporting, or continued schistosomiasis exposure in high-risk areas. Notably high prevalence was reported by recent studies such as Botes et al. [15] and Zacarias et al. [7], reinforcing this trend. Contrary to previous conclusions suggesting a post-2010 decline, our analysis instead indicates a rise in prevalence in the past decade, suggesting that global control efforts, while impactful, may be uneven or incomplete in certain regions [1,27,28].

One of the most notable findings of this review is the predominance of symptomatic, inflamed cases of SA. Among the 247 cases for which inflammation status was available, 91.9% were classified as inflamed, indicating that SA most often presents as clinically apparent appendicitis rather than an incidental histological finding. This reinforces its diagnostic and clinical relevance [5].

Regarding demographic factors, our analysis found no statistically significant association between sex and SA prevalence, despite some individual studies reporting male predominance [6,26]. The wide range in male-to-female ratios across studies (0.82-33.00) suggests heterogeneity likely driven by local population characteristics rather than a true global pattern.

The funnel plot revealed potential asymmetry, raising the possibility of publication bias or small-study effects. Several smaller studies reported higher prevalence estimates and lay outside the expected confidence limits, suggesting selective reporting or variability in study quality.

These findings carry several important implications. In endemic areas, routine histopathological examination of appendectomy specimens remains essential - not only for guiding patient care but also for epidemiological surveillance. In non-endemic countries, particularly those with large migrant populations, clinicians and pathologists should maintain a high index of suspicion for SA to avoid underdiagnosis [2].

At the policy level, the continued high prevalence in lower-middle-income and endemic countries calls for sustained investment in schistosomiasis control. This includes scaling up preventive chemotherapy, improving water and sanitation infrastructure, and ensuring equitable healthcare access, particularly in rural or underserved regions [29]. The increasing prevalence trend in recent years also signals the need for ongoing surveillance and prevention, as lapses in control efforts could lead to a resurgence in endemic zones [1,27].

This study has several limitations that should be considered when interpreting the findings. First, all included studies were observational in nature, which increases the risk of selection bias and limits causal inferences. Second, significant between-study heterogeneity was observed, likely reflecting differences in study design, population characteristics, diagnostic practices, and local schistosomiasis control efforts. Third, demographic data such as age and sex distribution were inconsistently reported, restricting subgroup analyses and limiting the ability to identify patient-level risk factors. Fourth, several studies did not clearly report their study periods or applied variable histopathological screening protocols, which may have influenced prevalence estimates. Fifth, underdiagnosis in non-endemic countries is possible due to lower clinical suspicion and inconsistent histopathological examination, suggesting that the true global burden of schistosomal appendicitis may be underestimated. Finally, although assessment for publication bias was undertaken, the presence of small-study effects cannot be excluded, and this may have influenced the pooled prevalence estimate.

## Conclusions

This review provides a robust global estimate of schistosomal appendicitis prevalence and confirms its continued clinical and epidemiological importance, especially in schistosomiasis-endemic regions. While prevalence is low globally, it remains significant in specific geographic and socioeconomic contexts. Routine histological analysis of appendectomy specimens should be maintained in high-risk areas, and global control strategies must continue to address the root causes of schistosomiasis to ultimately reduce its rare but important complications such as SA.
